# Sulphur *vs* NH Group: Effects on the CO_2_ Electroreduction Capability of Phenylenediamine-Cp Cobalt Complexes

**DOI:** 10.3390/molecules28052364

**Published:** 2023-03-04

**Authors:** Nicola Melis, Francesca Mocci, Annalisa Vacca, Luca Pilia

**Affiliations:** 1 Dipartimento di Ingegneria Meccanica, Chimica e dei Materiali (DIMCM), Università degli Studi di Cagliari, Via Marengo 2, 09123 Cagliari, Italy; 2 Dipartimento di Scienze Chimiche e Geologiche, Università di Cagliari, S.S. 554 km 4.500, 09042 Monserrato, Italy

**Keywords:** electrochemistry, reduction, CO_2_, cobalt complex, cyclic voltammetry, condensed Fukui function, DFT

## Abstract

The cobalt complex (**I**) with cyclopentadienyl and 2-aminothiophenolate ligands was investigated as a homogeneous catalyst for electrochemical CO_2_ reduction. By comparing its behavior with an analogous complex with the phenylenediamine (**II**), the effect of sulfur atom as a substituent has been evaluated. As a result, a positive shift of the reduction potential and the reversibility of the corresponding redox process have been observed, also suggesting a higher stability of the compound with sulfur. Under anhydrous conditions, complex **I** showed a higher current enhancement in the presence of CO_2_ (9.41) in comparison with **II** (4.12). Moreover, the presence of only one -NH group in **I** explained the difference in the observed increases on the catalytic activity toward CO_2_ due to the presence of water, with current enhancements of 22.73 and 24.40 for **I** and **II**, respectively. DFT calculations confirmed the effect of sulfur on the lowering of the energy of the frontier orbitals of **I**, highlighted by electrochemical measurements. Furthermore, the condensed Fukui function ***f ^−^*** values agreed very well with the current enhancement observed in the absence of water.

## 1. Introduction

Carbon dioxide (CO_2_), produced by both nature and human activities such as fossil fuel combustion and industrial processes, is the largest contributor among greenhouse gases to the well-known problem of global warming. On the other hand, CO_2_ is also considered as one of the most abundant and accessible carbon sources. Indeed, under appropriate conditions, it could be used as a fossil-free building block for the preparation of more complex derivatives and intermediates [1,2,3], even at lower costs, than those on the current market [4]. Therefore, for both environmental and economic reasons, catalysts able to reduce CO_2_ with high efficiency and selectivity are strongly desired [5].

Because of its high stability, the conversion of CO_2_ to compounds with the carbon atom in a lower oxidation state requires a large amount of energy to be achieved. Among other approaches, electrochemistry seems to be a very promising method for carbon dioxide reduction because of its versatility, simplicity, and cleanness [6,7,8,9,10,11]. Moreover, alternative and eco-sustainable methods for producing energy allow the use of electricity to become both economically and environmentally more sustainable and convenient. In fact, the cost of wind- and solar-generated electricity can be competitive with fossil fuel-based electricity generation technologies [12]. Furthermore, in periods of low consumption, the excess of electricity produced from renewable sources can be stored through electrochemical reactions, allowing overcoming disadvantages, such as variability and unpredictability, related to the above-mentioned renewable energy sources. From an electrochemical point of view, the reduction of CO_2_ is not thermodynamically favored due to the CO_2_-to-CO_2_^•−^ single-electron reduction step, which requires a high reductive potential to occur, mainly because of the large reorganization energy associated [8]. This reaction affects the global process with severe kinetics drawbacks despite the promising thermodynamic equilibrium potential values of other reduction products. In addition, other side reactions can be promoted and actively compete with CO_2_ reduction. For example, hydrogen evolution reaction (HER) is very favored in aqueous solvents and with most of the electrodes and conditions. For these reasons, there is an increasing interest in the development of suitable electrocatalysts that combine high activity and selectivity to improve the efficiency and selectivity towards a specific product [12,13,14].

Metal complex-based electrocatalysts are a wide class of catalysts that have been proved to be a good option in relation to the versatility and tunability of their properties by a suitable choice of the metal center and a proper ligand design [15,16,17,18,19,20]. Although CO_2_ is substantially considered a weak ligand due to its general chemical stability [21], several CO_2_−metal adducts have been isolated showing both electrophilic and nucleophilic characteristics [22]. The formation of such adducts is generally recognized as a first and necessary condition for the catalysis to take place: for this reason, a good choice of the ligand and the metal play a crucial role in the reactivity of these intermediates.

In this context, a biomimetic approach based on the use of “non-innocent” (redox-active) ligands could have a beneficial effect: in fact, in this kind of complexes, in addition to the metal, the ligand moiety also contributes to the redox activity of the molecular catalyst accepting and donating electrons [23,24,25,26,27,28,29,30]. This feature makes possible redox processes that involve multiple electrons also in complexes of metals which typically show one-electron redox activity (for example Fe, Cu, and Co), avoiding the use of catalysts based on precious and expensive metals such Pd, Pt, Rh, and Ir [31,32]. Moreover, in the specific case of CO_2_ reduction, the presence of “non-innocent” ligands in the molecule of the catalyst increases the selectivity toward CO_2_ reduction against HER. Indeed, the capability of “non-innocent” ligands to store electrons prevents a double reduction of the metal center which is necessary for the formation of the metal hydride [33,34]. Furthermore, the increased electron density on the reduced ligand can favor the stabilization of the adduct with CO_2_ via σ and π interactions [21,22,23,35].

Recently, we reported a study [36] on the homogeneous electrochemical reduction of CO_2_, using four heteroleptic complexes ([CpCo(R_n_L_NN_)]) of an Earth-abundant metal, such cobalt, bearing two “non-innocent” ligands: cyclopentadienyl (Cp) and R_n_-*o-*phenylenediamine (R_n_L_NN_) (n = 4, R = H or F; n = 1, R = *p*-COOH or *p*-NO_2_) [37,38,39,40]. Therein, we focused the attention on the effects of different kinds of electron-withdrawing substituents (-F, -COOH, and -NO_2_) on the phenylenediamine ring. The presence of these groups affects the electronic properties of the ligand, lowering the energy of the frontier orbitals and, consequently, facilitating the complex reduction. All the investigated compounds showed good electrocatalytic capability; in particular, the tetrafluorinated derivative proved to be the most effective one, with a 31-fold enhancement of the reduction current intensity in the presence of CO_2_ and water.

**Scheme 1:**
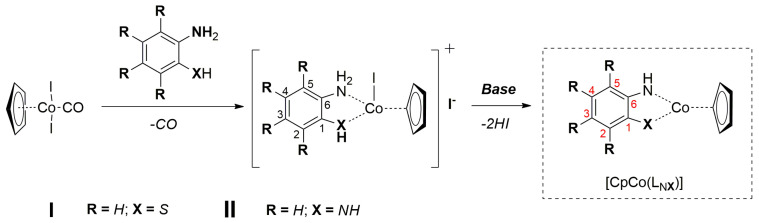
General synthetic route of complexes [CpCo(L_N**X**_)] **I** (X = S) and **II** (X = NH).

In this paper, we investigated the effect, induced by the replacement of one of the two amino groups of the phenylenediamine ligand with a sulfur atom, on the electrochemical and catalytic properties of this class of Co complexes. The replacement of a nitrogen donor with a bigger and more polarizable sulfur atom should affect the ligand’s redox-active characteristics, such as the efficiency in stabilizing charges and its capability as a supplier and/or storage of electrons [41,42,43,44]. Moreover, the presence of *d*-orbitals on the sulfur atom could play an important role on the catalyst−CO_2_ interactions and on the stability of such an adduct. Furthermore, in the literature, it has been pointed out that the presence of amino groups close to the metal center can play a key role in the overall reduction of CO_2_ [24,45,46,47]. These groups indeed, similar to the hydroxyl functionality [48,49,50], can act as a proton relay and stabilize the intermediates of the reaction by hydrogen bond interactions. For these reasons, we prepared the complex [CpCo(L_NS_)] (**I**), as summarized in Scheme 1, with the ligand 2-aminobenzenthiolate (L_NS_) which has one sulfur atom and a N-H group as donor units. This compound was experimentally studied by means of electrochemical techniques to establish its electrochemical behavior and its potential as an electrochemical catalyst for the CO_2_ reduction reaction. In addition, a computational study was performed by using the density functional theory calculations (DFT) [51] and the condensed Fukui function analysis [52], with the aim to investigate the electronic structure of this complex and to highlight the structure−properties relationship. To better highlight the sulfur atom effect, the results on compound **I** were discussed in comparison to those on complex [CpCo(L_NN_)] (**II**, L_NN_ = *o-*phenylenediamine).

## 2. Results and Discussion

**Figure 1:**
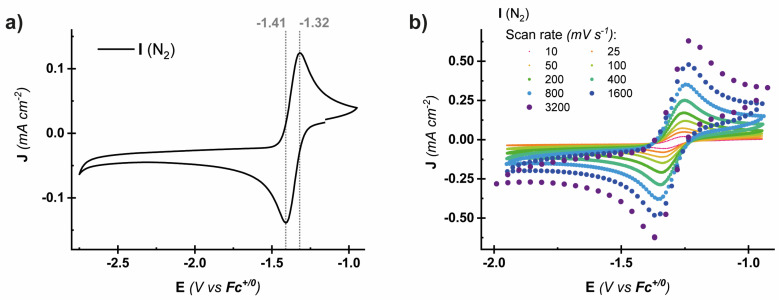
CVs of complex **I** under N_2_ recorded at 100 mV s^−1^ (**a**) and at different scan rates (10–3200 mV s^−1^ (**b**).

The electrochemical properties of compound **I** were investigated by CV techniques. The CV of **I** under an inert nitrogen atmosphere showed a redox couple centered at −1.36 V (Figure 1a), and the potential difference (ΔE) between the cathodic and anodic peaks was 90 mV at 100 mV s^−1^ of the scan rate. Table 1 resumes the peaks data related to **I** along with those of compound **II**. The reduction process of complex **I** occurred at less negative potentials than in the case of compound **II**, which showed this process at −1.74 V [36].

These data suggest that the presence of the sulfur atom in place of a N-H group stabilizes the anionic form of the catalyst.

Aiming at a broader characterization of the electrochemistry of compound **I**, CVs at different scan rates (0.01–3.2 V s^−1^) were recorded, as reported in Figure 1b.

The current intensities related to both the oxidation (I_ox_) and reduction (I_red_) processes showed a linear dependence on the square root of the scan rate (ν^1/2^), indicating an electron transfer process involving freely diffusing redox species (Figure S1), while the ratio between the two current intensities (I_ox_/I_red_) remained close to unity in all the investigated scan rates. Moreover, ΔE also increased linearly with the square root of the scan rate, pointing to a quasi-reversible redox process [53].

**Figure 2:**
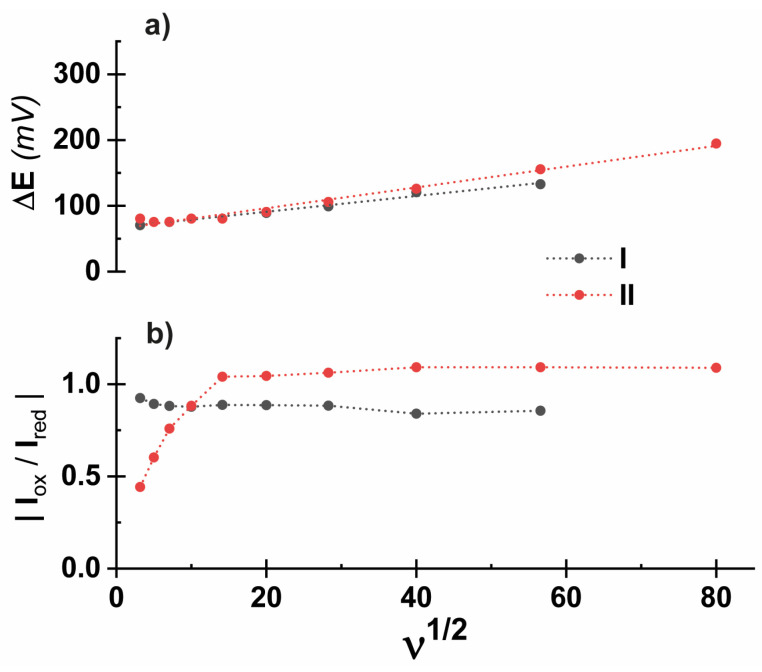
(**a**) ΔE vs. ν^1/2^; (**b**) |I_ox_/I_red_| vs. ν^1/2^.

For comparison purposes, the same electrochemical investigations were performed on complex **II**, which showed a similar behavior (Figure 2a). As can be seen in Figure 2b the ratio I_ox_/I_red_ was constant and close to 1 over the investigated range of scan rates for complex **I**, while for derivative **II** poor reversibility was observed at low scan rates which was regained at 200 mV s^−1^. From the analysis of these data, some information about chemical reactions eventually coupled with the reversible charge transfer process can be derived [53,54].

In fact, side chemical reactions that involve oxidized or reduced species can affect the long-term stability of the complex, depending on the kinetic and the reversibility/irreversibility of the reaction. As suggested by Nicholson [53], plotting the intensities ratio (I_ox_/I_red_) vs. ν^1/2^ (Figure 2b) can give important hints on kinetic features of side chemical reactions.

With regards to this aspect, the constant trend of complex **I** can be associated with a *quasi*-reversible electrochemical system with no subsequent chemical reaction (or a really slow one); thus, the ratio of I_ox_/I_red_ was close to 1 over the investigated scan rate range. Differently, in the case of complex **II**, the redox couple gained reversibility as the scan rate increased: in fact, as mentioned before, the current ratio reached 1 only at 200 mV s^−1^.

Such a behavior was consistent with a reversible electrochemical (E_R_) step followed by an irreversible chemical (C_I_) reaction (E_R_C_I_ mechanism) until a scan rate of 200 mV s^−1^, above which value the chemical process was suppressed because of the high scan rates and the system showed a quasi-reversible behavior. The rate constant k_f_ of the chemical step for compound **II** was determined as described in S.I. [53,55,56], obtaining a value of 1.90 s^−1^ (with t_1/2_ equals to 364.8 s). Since the side reaction of the reduced species could lead to a degradation of the complex, the behavior reported above seems to suggest a higher electrochemical stability of the sulphurated **I** derivative compared to that of its nitrogenated counterpart **II**.

**Figure 3:**
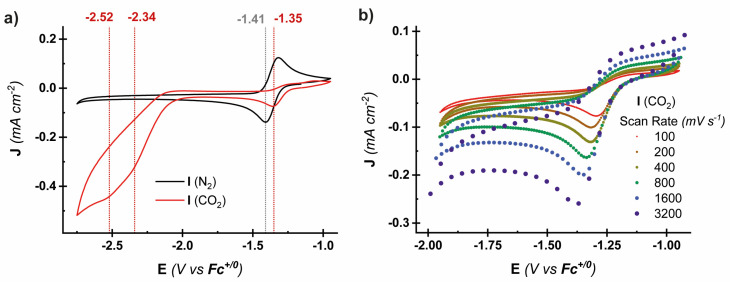
CVs of complex **I** under N_2_ (black line) and CO_2_ (red line) recorded at 100 mV s^−1^ (**a**) and under CO_2_ at different scan rates (100–3200 mV s^−1^) (**b**).

The electrochemical properties of complex **I** as a catalyst were tested with CV measurements in a CO_2_-saturated solution. As it can be observed in Figure 3a, in the presence of CO_2_, a faradaic cathodic current was well visible starting from −2.04 V, correlated to the electrochemical carbon dioxide reduction process. Furthermore, as previously pointed out in similar cases, an anodic shift (≈60 mV) of the reduction peak was observed, while a quasi-reversible redox couple disappeared. It is interesting to point out that, as already observed in the other studied examples of this class of compounds, the current density of the reduction peaks under a CO_2_ environment was lower in comparison to under a N_2_ atmosphere: this aspect can be due to the formation of a [CoCpL-CO_2_] adduct with a different electrochemical behavior, diminishing the concentration of the free complex species.

Although not clearly shaped as peaks, the current onset of the reduction branch of the CV started at ca. −2.0 V and presented two small shoulders at −2.34 V and −2.52 V; therefore, these potentials were chosen for evaluating the catalytic activity of **I**. As summarized in Table 2, compared with the corresponding values observed with N_2_, a current 7.16- and 9.41-fold higher were observed at −2.34 V and −2.52 V, respectively; these enhancements can be related to the ability of the complex to catalyze the CO_2_ reduction. In fact, when the CV was performed without the complex in both N_2_- and CO_2_-saturated solutions, no significant increase in current values were observed, indicating that the CO_2_ reduction did not occur in a substantial manner in the investigated range of the potential (see Figure S3). It is interesting to note that in the case of complexes **II** the presence of CO_2_ increased the cathodic current by 4.12-fold with respect to that with N_2_ [36], suggesting a higher catalytic activity of compound **I**.

The electrochemistry of **I** was investigated in the presence of CO_2_ with a series of CVs performed at different scan rates (0.1–3.2 V s^−1^) as reported in Figure 3b. As it became more noticeable by normalizing the current values for the square root of the scan rates (Figure S4), it seems that just a minimum portion of the reoxidation process was regained, increasing the scan rate. This behavior should indicate a very fast chemical irreversible step following CO_2_ binding. Similar cases have been reported in relation to very fast processes such as isomerization or protonation [57]. Nevertheless, since the current intensity varied linearly with ν^1/2^, the reduction of complex **I** appeared to be a diffusion-controlled process [36].

As with **I** and also with **II**, the process involving CO_2_ was too fast to be reversed in these conditions, and no oxidation peak can be observed, even at very high scan rates (>10 V s^−1^). Thus, under a CO_2_ atmosphere, the mono-reduced species appeared to be more reactive with respect to under inert N_2_ conditions for both the derivatives.

**Figure 4:**
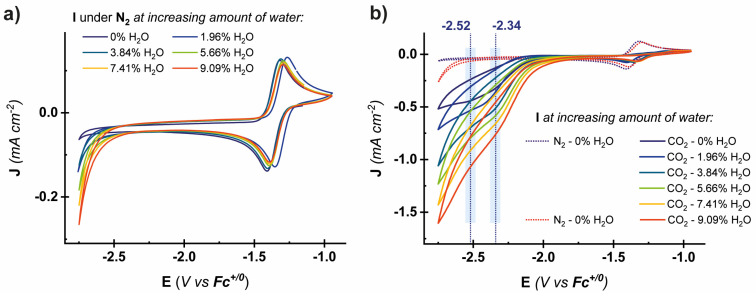
CVs of complex **I** under N_2_ (**a**) and under CO_2_ (**b**) at different water amounts.

It is well-known that the presence of proton-coupled electron transfer can enhance the catalytic capability of several molecular catalysts [47,48,58,59]. For this reason, the electrochemical behaviors of complex **I** and its catalytic response under CO_2_ have been also investigated in the presence of an increasing amount of water. Therefore, we registered the CV upon successive additions of 200 µL of water to an anhydrous DMSO solution containing the complex in both N_2_ (Figure 4a) and CO_2_ (Figure 4b) atmospheres: the maximum value of 1 mL of water was added, corresponding to 9.09%_v/v_. It is expected that a catalytic current enhancement with an increasing amount of water under a nitrogen atmosphere can be related to HER process. It is worth noting that, in the case of complex **I**, the current enhancement observed with N_2_ was largely smaller than that measured when the solution and the atmosphere were saturated with carbon dioxide, even in the presence of a large amount of water. These findings suggest a high selectivity of this catalyst toward the CO_2_ reduction over proton reduction [36].

In Table 3, a comparison between the current enhancements (**I**_CO2_/I_N2_) upon 9.09%_v/v_ water additions, calculated at two different potentials (−2.34 V and −2.52 V) under both N_2_ and CO_2_ atmospheres, is reported. Comparing the I_CO2_/I_N2_ ratios in the presence of 9.09% of H_2_O of compound **I** (16.89 and 22.73), with those of complex **II** (24.40), it comes to light that, although **I** seems to be the most effective catalyst in anhydrous conditions, in the presence of water it shows a slightly smaller current enhancement with respect to **II**.

These different performances could be explained by considering the role played by the molecules of water in reactions with catalysts that present amino functionalities. In fact, it has been proposed that the molecules of H_2_O can form H-bonds with the amino group and the CO_2_ in the adduct with the catalyst; these interactions stabilize the intermediates of the reaction, favoring the CO_2_ reduction process [24,45,46,47]. As far as our study is concerned, in complex **I** the ligand has only one N-H group, whereas the phenylenediamine ligand (L_NN_) presents two of those. Therefore, it is possible that in the case of compound **I**, the substitution of the NH group with the S atom implicates a smaller stabilizing effect due to a lack of H-bonds on the intermediates, causing, in the presence of water, a decrease in the catalytic activity of this complex in comparison with that of compound **II**. Moreover, Table 3 also reports the relative turnover frequency (R-TOF), (I_cat_/I_p_)^2^, which is proportional to the TOF and a convenient guideline for an easier and more direct comparison between the catalytic performances of similar molecules.

For the calculation of these quantities, we considered the current intensities under a CO_2_ atmosphere for both the chosen potentials (I_cat_), but due to the lack of the substrate (CO_2_), no peak was observed in potentials more negative than −1.6 V in non-catalytic conditions. Thus, following the example of Artero [45], the current intensity under N_2_ of the one-electron reduction peak R1 at the corresponding content of water was considered as I_p_. Indeed, relative TOF values reflect the current enhancement trend: thus, as expected, **II** presented the highest value (280.07 vs 83.90 s^−1^ at a similar potential) in the presence of water, whereas in anhydrous conditions the highest value was generated in the case of **I**.

It is interesting to notice that, contrary to its *N*,*N* counterparts, this *N*,*S* complex seemed to preserve the reversibility of the redox couple at around −1.32 V, even in the presence of water. This feature suggests that in the case of **I**, the proton reduction—if any—does not involve the catalyst re-oxidation (see Figure 4a).

**Figure 5:**
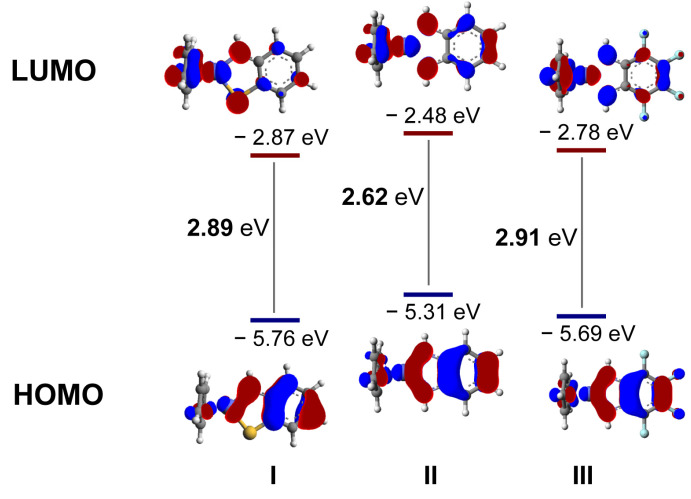
HOMO and LUMO for the neutral derivatives **I** and **II** and their corresponding energies and gaps.

In order to highlight the effect of the sulfur atom on the electronic structure of this family of complexes, DFT calculations at the B3LYP/6-311+G(d,p) level were performed on compounds **I** and **II**. Figure 5 depicts the frontier molecular orbitals (FOs) with the corresponding energies for the two compounds in their neutral form. These results suggest that, in comparison with complex **II**, the presence of a sulfur atom lowers both the highest occupied molecular orbital (HOMO) and the lowest unoccupied molecular orbital (LUMO) energies for complex **I**.

The trend in the LUMOs energies agreed with the trend observed in the experimental redox potentials: indeed, complex **I** showed the easiest reduction process, whereas compound **II** required the most negative potential to be reduced. Moreover, the reduction potential value predicted by applying the empirical relation proposed by D’Andrade et al., which correlates the LUMO energy (E_LUMO_) and the reduction potential (E_red_), was also consistent with the measured potential (−1.24 V in the case of **I**) [60].

**Figure 6:**
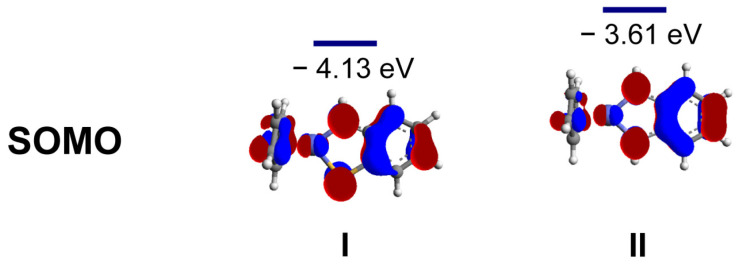
SOMO and corresponding energies of compounds **I** and **II** in their anionic reduced form.

Figure 6 shows the single occupied molecular orbital (SOMO) of the same compounds in their monoreduced form as radical anions. As one can see, the observed trend was consistent with the HOMO/LUMO energies, and complex **I** showed the SOMO with the lowest energy, accordingly with its capability to better stabilize the extra negative charge due to the presence of the sulfur atom in comparison with complexes **II**.

As far as the frontier orbitals’ shapes are concerned, some differences can be observed between the FOs of complexes **I** and **II**; indeed, this comparison highlights the effect of the sulfur atom on the electronic structure of this compound. In fact, looking at the benzene ring in the case of HOMOs, while atoms C2 and C5 (see Scheme 1) give very similar and small contributions to the MO in **II,** in **I,** C2 and C5 orbitals contribute in a negligible and significant manner, respectively. Furthermore, no sulfur orbitals contribute to the HOMO of **I**, whereas nitrogen orbitals contribute in a sizable way to it. Considering the LUMO, carbon atoms 2, 3, 4, and 5 contribute almost in the same manner to this orbital in the case of complexes with the phenylenediamine ligand. In the case of **I,** however, the most important contributions to the benzene ring come from atoms C1, C3, and C5. These differences are a clear effect of the non-symmetry of the ligand, when a sulfur atom replace a nitrogen: therefore, the impacts of the sulfur atom on the electronic distributions of ortho- and para-positions seem to prevail on the one of the amine group. Moreover, for both FOs in all these complexes, important contributions are made by the Cp ring and the cobalt *d* orbitals.

The reactivity of **I** and **II** compounds was further investigated by calculating the atomic dipole-corrected Hirshfeld atomic charge (ADHC) [61] on each atom (either in the optimized reduced form of the complexes and in the neutral form with the same geometry of the reduced form) and the corresponding value of the condensed Fukui ***f***–function (see Table 4). From the atomic charges of the anionic species, it can be noticed that the charge on the sulfur atom was more than 25% higher than that on the nitrogen atom. The ***f***–values indicates that the contribution to electron donation of the nitrogen atoms was ca. 0.08 e^−^ in both **I** and **II**, and that of sulfur was much higher (ca. 0.23 e^−^); this indicated that the electrophilic attack on the reduced form of **I** was likely to occur on sulfur. Such a difference between the heteroatoms in the ligand correlated with the increased catalytic capability of compound **I**, reported in Table 2.

Considering all of the atoms in the catalytic core comprising the metal and the surrounding heavy atoms (i.e., N + S + Co + Cp in **I** and 2 N + Co + Cp in **II**), it can be noticed that while the ADCH charge of the core of the reduced species did not correlate with the catalytic capability reported in Table 2, the ***f***–values did correlate. Specifically, the order of the ***f***–values (**I** > **II**) was in agreement with the experimental current enhancement reported in Table 2.

## 3. Materials and Methods

### 3.1. Synthesis

All the reagents and solvents were purchased from Sigma-Aldrich (St. Louis, MO, USA) and used as received. The intermediate cobalt complex CpCoI_2_CO was prepared as described by King [62]. The synthesis of complexes **I** and **II** were performed as previously reported by Heck [40,63].

### 3.2. Electrochemical Methods

The electrochemical properties of complex **I** were investigated by the cyclic voltammetry (CV) technique both under nitrogen and carbon dioxide atmospheres, using different scan rates from 0.010 up to 12.8 V s^−1^. All the measurements were carried out with a three-electrode cell using glassy carbon (3 mm in diameter) as a working electrode, a platinized titanium net as a counter electrode, and a platinum wire as a pseudo-reference electrode. The electrochemical experiments were performed at room temperature using an AUTOLAB PGSTAT302N (Metrohm, Switzerland) potentiostat/galvanostat controlled with the NOVA software. Anhydrous dimethylsulfoxide, DMSO (Sigma-Aldrich), used as received, was chosen as a model solvent mainly for its typical high boiling point, which should reasonably avoid any changes in concentration by the evaporation of the solvent during nitrogen and CO_2_ insufflation processes. The supporting electrolyte was 0.1 M [*n*Bu_4_N][PF_6_] (Sigma-Aldrich); in these conditions, the redox couple Fc^0/+^ was centered at +0.45 V. The working electrode was cleaned before each measurement as follows: after extensive washing with distilled water and isopropanol, the glassy carbon electrode was polished with diamond suspensions (0.05, 0.25, 1, and 3 μm) and a wet pad.

Depending on the runs, the solutions containing complex **I** were bubbled with N_2_ or CO_2_ for 30 min. The effect of the presence of water was measured by an incremental addition of degassed deionized H_2_O in an electrochemical solution by an air-tight syringe.

The number of electrons involved in the reversible redox processes were calculated by using the following Equation (1):

(1) $$\left|E_{p}-E_{\frac{p}{2}}\right|=\frac{59\;\mathrm{mV}}{n}$$

### 3.3. Computational Methods

The structure of the studied compounds in both the neutral and anionic radical forms was optimized by means of DFT calculations. In detail, geometry optimization was performed by employing the B3LYP functional as implemented in the commercially available suite of programs Gaussian 16 [64] using the 6-311+G(d,p) basis set for all atoms. The solvent effects on the geometry and on the electronic properties were included by further optimizing the in vacuo global minimum of each species using the current implementation in Gaussian 16 of the polarizable conductor-like continuum model (CPCM) [65,66] for the DMSO solvent. All PCM calculations were carried out at 298.15 K, and the molecular cavity was constructed using the default procedure in Gaussian 16.

Graphics of molecular models were generated using ArgusLab 4.0 [67]. Atomic dipole moment-corrected Hirshfeld population (ADCH) charges were calculated using the software Multiwfn [68]. The condensed Fukui function, which can be calculated from the ADCH charges, was extensively used to predict reactive sites. The Fukui condensed function for the electrophilic attack (*f* ^−^) was calculated as:

(2) $$f_{k}^{-}=q_{k}\left(N\right)-q_{k}\left(N-1\right)$$

where *q_k_*(*N*) is the charge at the atomic center *k* calculated on the optimized geometry of the reduced complex, and *q_k_*(*N – 1*) is the charge of the corresponding neutral form with the same geometry, both calculated at the CPCM(DMSO)/UB3LYP/6-311+g(d,p) level. The electrophilic attack is likely to occur on the atoms with larger *f_k_* ^−^.

## 4. Conclusions

In this paper, the effect of the replacement of a NH group with a sulfur atom in cyclopentadienyl phenylenediamine cobalt complexes towards electrochemical CO_2_ reduction has been evaluated.

As expected, a more polarizable atom such as sulfur, with empty *d* orbitals, is likely to stabilize more effectively an extra charge as in the case of the monoreduced radical anion derivative formation. In the context of metal complex-mediated electroreduction of CO_2_, the effect of replacing two opposite nitrogen atoms with sulfur in the Ni(cyclam) complex has been highlighted. As pointed out by Gerschel et al., such a substitution leads to lower overpotentials for the reduction of CO_2_, of which the reduction would occur at a more positive potential, and to enhance the HER capability of the complex [69]. Moreover, in the field of photocatalyzed reduction, Kojima and his research group proved the selectivity of a Ni^II^-based N_2_S_2_-type complex for reduction of CO_2_ to CO, reporting a higher TON value for nickel complexes in that photocatalytic setup [70]. This aspect of sulfur atom reactivity is reflected on the reversibility of the characteristic redox couple of **I**, which was constant on all the investigated scan rates. These features of complex **I** had an effect on its catalytic properties; indeed, it presented a higher I_CO2_/I_N2_ ratio under anhydrous conditions with respect to its nitrogenated counterpart **II**. Moreover, the higher reversibility is also a hint of the higher stability of complex **I** in comparison with **II**. However, in the presence of water, compound **I** showed a slightly smaller I_CO2_/I_N2_ in comparison with **II**. This result can be related to the presence of only one nitrogen atom; in fact, the NH group favors catalyst−H_2_O interactions which play an important role in the CO_2_ reduction process. Moreover, the different behavior of complex **I** with water seemed to be confirmed by the presence of the re-oxidation process observed, when CVs were carried out under N_2_ in the presence of water. This fact might suggest a low affinity of **I** for hydrogen reduction, and therefore, it might be a hint of a high selectivity towards CO_2_.

The experimental results were supported by computational investigations. DFT calculations confirmed the effect of sulfur on the lowering of the energy of the FOs and, consequently, the reduction potential of **I**. The sulfur effect on stabilizing the extra charge has been highlighted both by the analysis of the atomic charges and by the calculated energies of the SOMO for the corresponding radical anions of complexes **I** and **II**. Interestingly, the current enhancement observed in the absence of water correlated very well with the probability of an electrophilic attack on the core atoms of the complexes, as measured by the condensed Fukui function ***f*^−^**.

## Figures and Tables

**Table 1:** Peaks analyses of complexes **I** and **II** under an inert nitrogen atmosphere.

	O1 (V)	R1 (V)	E_1/2_ (V)	ΔE (mV)
**I**	−1.32	−1.41	−1.36	90
**II**	−1.66	−1.74	−1.70	80

**Table 2:** Peaks analyses of complexes **I** and **II** in CO_2_-saturated solutions.

	R1 (V)	E_p/2_ (V)	R2 (V)	E (V)	I_CO2_/I_N2_
**I**	−1.35	−1.29	−2.34	−2.34	7.16
			−2.52	−2.52	9.41
**II**	−1.78	−1.72	−2.52	−2.54	4.12

**Table 3:** I_CO_^2^/I_N_^2^ and (I_cat_/I_p_)^2^ values summary for complexes **I** and **II** under anhydrous conditions and in the presence of water calculated at the corresponding potentials.

	I				II	
E_cat_ (V)	−2.34		−2.52		−2.54	
**H_2_O** (%_v/v_)	0	9.09	0	9.09	0	9.09
**I_CO2_/I_N2_**	7.16	16.89	9.41	22.73	4.12	24.40
**(I_cat_/I_p_)** ^2^	5.48	42.25	10.26	83.90	3.32	280.07
**I_HER_/I** ^[a]^		1.02		1.37		1.09

^[a]^ Calculated at 9.09%_v/v_ of water after reconditioning the system with nitrogen.

**Table 4:** ADCH charges for the core group atoms of the catalyst in the reduced form (Red^•–^) and in the neutral species (Neutr)—both calculated on the optimized anion and the corresponding condensed Fukui function (*f* ^−^).

	I			II		
	Red^−^	Neutr	*f* ^−^	Red^−^	Neutr	*f* ^−^
**Co**	−0.105	−0.097	−0.008	−0.136	−0.077	−0.059
**N**	−0.326	−0.241	−0.086	−0.350	−0.266	−0.083
**C (Cp)**	−0.159	−0.105	−0.054	−0.169	−0.131	−0.038
**S**	−0.455	−0.228	−0.226			
**Sum**	−1.682	−1.091	−0.591	−1.682	−1.267	−0.415

## Data Availability

Not applicable.

## Supplementary Materials

The following supporting information can be downloaded at: https://www.mdpi.com/article/10.3390/molecules28052364/s1, Figure S1: Plots of oxidation (red) and reduction (blue) current densities vs. (ν)^1/2^ for complex **I**; Figure S2: Graph E_p_-E_1/2_ vs. ln(1/ν) for derivative **II**; Figure S3: CVs of TBAPF6/DMSO (0.1 M) under N_2_ (solid blue line) and CO_2_ (solid red line). CVs of complex **I** under the same conditions were reported for comparisons (dotted lines); Figure S4: CVs of complex **I** at different scan rates. Current densities J were normalized by the square root of the scan rate; Figure S5: CVs of complex **II** at different scan rates.

## References

[1] Sakakura T., Choi J.-C., Yasuda H. (2007). Transformation of Carbon Dioxide. Chem. Rev..

[2] Liu Q., Wu L., Jackstell R., Beller M. (2015). Using Carbon Dioxide as a Building Block in Organic Synthesis. Nat. Commun..

[3] Abanades J.C., Rubin E.S., Mazzotti M., Herzog H.J. (2017). On the Climate Change Mitigation Potential of CO_2_ Conversion to Fuels. Energy Environ. Sci..

[4] Bushuyev O.S., de Luna P., Dinh C.T., Tao L., Saur G., van de Lagemaat J., Kelley S.O., Sargent E.H. (2018). What Should We Make with CO_2_ and How Can We Make It?. Joule.

[5] Wang M., Torbensen K., Salvatore D., Ren S., Joulié D., Dumoulin F., Mendoza D., Lassalle-Kaiser B., Işci U., Berlinguette C.P. (2019). CO_2_ Electrochemical Catalytic Reduction with a Highly Active Cobalt Phthalocyanine. Nat. Commun..

[6] Lote D.A. (2014). Literature Survey on Electrochemical Reduction of CO_2_. Int. J. Electron. Electr. Eng..

[7] Costentin C., Robert M., Savéant J.-M. (2013). Catalysis of the Electrochemical Reduction of Carbon Dioxide. Chem. Soc. Rev..

[8] Benson E.E., Kubiak C.P., Sathrum A.J., Smieja J.M. (2009). Electrocatalytic and Homogeneous Approaches to Conversion of CO_2_ to Liquid Fuels. Chem. Soc. Rev..

[9] Orella M.J., Román-Leshkov Y., Brushett F.R. (2018). Emerging Opportunities for Electrochemical Processing to Enable Sustainable Chemical Manufacturing. Curr. Opin. Chem. Eng..

[10] Seh Z.W., Kibsgaard J., Dickens C.F., Chorkendorff I., Nørskov J.K., Jaramillo T.F. (2017). Combining Theory and Experiment in Electrocatalysis: Insights into Materials Design. Science.

[11] Li F., Mocci F., Zhang X., Ji X., Laaksonen A. (2021). Ionic Liquids for CO_2_ Electrochemical Reduction. Chin. J. Chem. Eng..

[12] Franco F., Fernández S., Lloret-Fillol J. (2019). Advances in the Electrochemical Catalytic Reduction of CO2 with Metal Complexes. Curr. Opin. Electrochem..

[13] Ishida H. (2018). Electrochemical/Photochemical CO_2_ Reduction Catalyzed by Transition Metal Complexes. Carbon Dioxide Chemistry, Capture and Oil Recovery.

[14] Zhang Z., Vasiliu T., Li F., Laaksonen A., Mocci F., Ji X. (2021). Electrochemically Driven Efficient Enzymatic Conversion of CO_2_ to Formic Acid with Artificial Cofactors. J. CO2 Util..

[15] Collin J.P., Sauvage J.P. (1989). Electrochemical Reduction of Carbon Dioxide Mediated by Molecular Catalysts. Coord. Chem. Rev..

[16] Savéant J.M. (2008). Molecular Catalysis of Electrochemical Reactions. Mechanistic Aspects. Chem. Rev..

[17] Francke R., Schille B., Roemelt M. (2018). Homogeneously Catalyzed Electroreduction of Carbon Dioxide—Methods, Mechanisms, and Catalysts. Chem. Rev..

[18] Windle C.D., Reisner E. (2015). Heterogenised Molecular Catalysts for the Reduction of CO_2_ to Fuels. CHIMIA Int. J. Chem..

[19] Eisenberg R., Hendriksen D.E. (1979). The Binding and Activation of Carbon Monoxide, Carbon Dioxide, and Nitric Oxide and Their Homogeneously Catalyzed Reactions. Advances in Catalysis.

[20] Mascetti J., Arasta M. (2010). Carbon Dioxide Coordination Chemistry and Reactivity of Coordinated CO_2_. Carbon Dioxide as Chemical Feedstock.

[21] Smieja J.M., Sampson M.D., Grice K.A., Benson E.E., Froehlich J.D., Kubiak C.P. (2013). Manganese as a Substitute for Rhenium in CO_2_ Reduction Catalysts: The Importance of Acids. Inorg. Chem..

[22] Sampson M.D., Nguyen A.D., Grice K.A., Moore C.E., Rheingold A.L., Kubiak C.P. (2014). Manganese Catalysts with Bulky Bipyridine Ligands for the Electrocatalytic Reduction of Carbon Dioxide: Eliminating Dimerization and Altering Catalysis. J. Am. Chem. Soc..

[23] Hartl F., Rosa P., Ricard L., le Floch P., Záliš S. (2007). Electronic Transitions and Bonding Properties in a Series of Five-Coordinate “16-Electron” Complexes [Mn(CO)3(L2)]− (L2=chelating Redox-Active π-Donor Ligand). Coord. Chem. Rev..

[24] Song J., Klein E.L., Neese F., Ye S. (2014). The Mechanism of Homogeneous CO_2_ Reduction by Ni(Cyclam): Product Selectivity, Concerted Proton–Electron Transfer and C–O Bond Cleavage. Inorg. Chem..

[25] Hu X.M., Rønne M.H., Pedersen S.U., Skrydstrup T., Daasbjerg K. (2017). Enhanced Catalytic Activity of Cobalt Porphyrin in CO_2_ Electroreduction upon Immobilization on Carbon Materials. Angew. Chem. Int. Ed..

[26] Lacy D.C., McCrory C.C.L., Peters J.C. (2014). Studies of Cobalt-Mediated Electrocatalytic CO_2_ Reduction Using a Redox-Active Ligand. Inorg. Chem..

[27] Shimoda T., Morishima T., Kodama K., Hirose T., Polyansky D.E., Manbeck G.F., Muckerman J.T., Fujita E. (2018). Photocatalytic CO_2_ Reduction by Trigonal-Bipyramidal Cobalt(II) Polypyridyl Complexes: The Nature of Cobalt(I) and Cobalt(0) Complexes upon Their Reactions with CO_2_, CO, or Proton. Inorg. Chem..

[28] Nakada A., Matsumoto T., Chang H.-C. (2022). Redox-Active Ligands for Chemical, Electrochemical, and Photochemical Molecular Conversions. Coord. Chem. Rev..

[29] Sutradhar M., Pombeiro A.J.L., da Silva J.A.L. (2021). Water Oxidation with Transition Metal Catalysts with Non-Innocent Ligands and Its Mechanisms. Coord. Chem. Rev..

[30] Tarrago M., Ye S., Neese F. (2022). Electronic Structure Analysis of Electrochemical CO_2_ Reduction by Iron-Porphyrins Reveals Basic Requirements for Design of Catalysts Bearing Non-Innocent Ligands. Chem. Sci..

[31] Chirik P.J., Wieghardt K. (2010). Radical Ligands Confer Nobility on Base-Metal Catalysts. Science.

[32] Praneeth V.K.K., Ringenberg M.R., Ward T.R. (2012). Redox-Active Ligands in Catalysis. Angew. Chem. Int. Ed..

[33] Benson E.E., Sampson M.D., Grice K.A., Smieja J.M., Froehlich J.D., Friebel D., Keith J.A., Carter E.A., Nilsson A., Kubiak C.P. (2013). The Electronic States of Rhenium Bipyridyl Electrocatalysts for CO_2_ Reduction as Revealed by X-Ray Absorption Spectroscopy and Computational Quantum Chemistry. Angew. Chem. Int. Ed..

[34] Grice K.A., Saucedo C. (2016). Electrocatalytic Reduction of CO_2_ by Group 6 M(CO)_6_ Species without “Non-Innocent” Ligands. Inorg. Chem..

[35] Bourrez M., Molton F., Chardon-Noblat S., Deronzier A. (2011). [Mn(Bipyridyl)(CO)_3_Br]: An Abundant Metal Carbonyl Complex as Efficient Electrocatalyst for CO_2_ Reduction. Angew. Chem. Int. Ed..

[36] Melis N., Mocci F., Vacca A., Pilia L. (2020). Novel Homogeneous Selective Electrocatalysts for CO _2_ Reduction: An Electrochemical and Computational Study of Cyclopentadienyl-Phenylendiamino-Cobalt Complexes. Sustain. Energy Fuels.

[37] Kaim W. (2011). Manifestations of Noninnocent Ligand Behavior. Inorg. Chem..

[38] Kaim W. (2012). The Shrinking World of Innocent Ligands: Conventionaland Non-Conventional Redox-Active Ligands. Eur. J. Inorg. Chem..

[39] Fourmigué M., Cauchy T., Nomura M. (2009). Experimental and Theoretical Evaluation of Magnetic Coupling in Organometallic Radicals: The Eloquent Case of Face-to-Face Cp⋯Cp Interactions. CrystEngComm.

[40] Pilia L., Shuku Y., Dalgleish S., Hofmann D.W.M., Melis N., Awaga K., Robertson N. (2020). Effect of Fluorination on the Crystal and Electronic Structure of Organometallic Cyclopentadienyl-Phenylenediamino-Cobalt Complexes. J. Organomet. Chem..

[41] Spielvogel K.D., Coughlin E.J., Petras H., Luna J.A., Benson A., Donahue C.M., Kibasa A., Lee K., Salacinski R., Bart S.C. (2019). The Influence of Redox-Innocent Donor Groups in Tetradentate Ligands Derived from *o* -Phenylenediamine: Electronic Structure Investigations with Nickel. Inorg. Chem..

[42] Sarkar P., Sarmah A., Mukherjee C. (2022). Where Is the Unpaired Electron Density? A Combined Experimental and Theoretical Finding on the Geometric and Electronic Structures of the Co(III) and Mn(IV) Complexes of the Unsymmetrical Non-Innocent Pincer ONS Ligand. Dalton Trans..

[43] Combariza M.Y., Vachet R.W. (2002). Gas-Phase Ion-Molecule Reactions of Transition Metal Complexes: The Effect of Different Coordination Spheres on Complex Reactivity. J. Am. Soc. Mass Spectrom..

[44] Martin R.B. (2002). Practical Hardness Scales for Metal Ion Complexes. Inorg. Chim. Acta.

[45] Roy S., Sharma B., Pécaut J., Simon P., Fontecave M., Tran P.D., Derat E., Artero V. (2017). Molecular Cobalt Complexes with Pendant Amines for Selective Electrocatalytic Reduction of Carbon Dioxide to Formic Acid. J. Am. Chem. Soc..

[46] Schneider J., Jia H., Kobiro K., Cabelli D.E., Muckerman J.T., Fujita E. (2012). Nickel(Ii) Macrocycles: Highly Efficient Electrocatalysts for the Selective Reduction of CO_2_ to CO. Energy Environ. Sci..

[47] Chapovetsky A., Do T.H., Haiges R., Takase M.K., Marinescu S.C. (2016). Proton-Assisted Reduction of CO_2_ by Cobalt Aminopyridine Macrocycles. J. Am. Chem. Soc..

[48] Costentin C., Passard G., Robert M., Savéant J.-M. (2014). Pendant Acid–Base Groups in Molecular Catalysts: H-Bond Promoters or Proton Relays? Mechanisms of the Conversion of CO_2_ to CO by Electrogenerated Iron(0)Porphyrins Bearing Prepositioned Phenol Functionalities. J. Am. Chem. Soc..

[49] Costentin C., Passard G., Savéant J.M. (2015). Benchmarking of Homogeneous Electrocatalysts: Overpotential, Turnover Frequency, Limiting Turnover Number. J. Am. Chem. Soc..

[50] Costentin C., Drouet S., Robert M., Savéant J.M. (2012). A Local Proton Source Enhances CO_2_ Electroreduction to CO by a Molecular Fe Catalyst. Science.

[51] Parr R.G., Yang W. (1989). Density Functional Theory of Atoms and Molecules. Density Functional Theory of Atoms and Molecules.

[52] Parr R.G., Yang W. (1984). Density Functional Approach to the Frontier-Electron Theory of Chemical Reactivity. J. Am. Chem. Soc..

[53] Nicholson R.S., Shain I. (1964). Theory of Stationary Electrode Polarography. Anal. Chem..

[54] Costentin C., Savéant J.-M. (2014). Multielectron, Multistep Molecular Catalysis of Electrochemical Reactions: Benchmarking of Homogeneous Catalysts. ChemElectroChem.

[55] Hopkins Leseberg J.A., Lionetti D., Day V.W., Blakemore J.D. (2021). Electrochemical Kinetic Study of [Cp*Rh] Complexes Supported by Bis(2-Pyridyl)Methane Ligands. Organometallics.

[56] Gonell S., Assaf E.A., Duffee K.D., Schauer C.K., Miller A.J.M.M. (2020). Kinetics of the Trans Effect in Ruthenium Complexes Provide Insight into the Factors That Control Activity and Stability in CO_2_ Electroreduction. J. Am. Chem. Soc..

[57] Froehlich J.D., Kubiak C.P. (2015). The Homogeneous Reduction of CO_2_ by [Ni(Cyclam)]+: Increased Catalytic Rates with the Addition of a CO Scavenger. J. Am. Chem. Soc..

[58] Chapovetsky A., Welborn M., Luna J.M., Haiges R., Miller T.F., Marinescu S.C. (2018). Pendant Hydrogen-Bond Donors in Cobalt Catalysts Independently Enhance CO_2_ Reduction. ACS Cent. Sci..

[59] Jakobsen J.B., Rønne M.H., Daasbjerg K., Skrydstrup T. (2021). Are Amines the Holy Grail for Facilitating CO_2_ Reduction?. Angew. Chem. Int. Ed..

[60] D’Andrade B.W., Datta S., Forrest S.R., Djurovich P., Polikarpov E., Thompson M.E. (2005). Relationship between the Ionization and Oxidation Potentials of Molecular Organic Semiconductors. Org. Electron..

[61] Lu T., Chen F. (2012). Atomic Dipole Moment Corrected Hirshfeld Population Method. J. Theor. Comput. Chem..

[62] King R.B. (1966). Organometallic Chemistry of the Transition Metals. XI. Some New Cyclopentadienyl Derivatives of Cobalt and Rhodium. Inorg. Chem..

[63] Heck R.F. (1968). Cyclopentadienylcobalt Derivatives of Chelating Aromatic Ligands. Inorg. Chem..

[64] Frisch M.J., Trucks G.W., Schlegel H.B., Scuseria G.E., Robb M.A., Cheeseman J.R., Scalmani G., Barone V., Petersson G.A., Nakatsuji H. (2016). Gaussian 16, Rev. A.01.

[65] Barone V., Cossi M. (1998). Quantum Calculation of Molecular Energies and Energy Gradients in Solution by a Conductor Solvent Model. J. Phys. Chem. A.

[66] Cossi M., Rega N., Scalmani G., Barone V. (2003). Energies, Structures, and Electronic Properties of Molecules in Solution with the C-PCM Solvation Model. J. Comput. Chem..

[67] Thompson M.A. ArgusLab 4.0.1. http://www.arguslab.com/arguslab.com/ArgusLab.html.

[68] Lu T., Chen F. (2012). Multiwfn: A Multifunctional Wavefunction Analyzer. J. Comput. Chem..

[69] Gerschel P., Warm K., Farquhar E.R., Englert U., Reback M.L., Siegmund D., Ray K., Apfel U.-P. (2019). Sulfur Substitution in a Ni(Cyclam) Derivative Results in Lower Overpotential for CO_2_ Reduction and Enhanced Proton Reduction. Dalton Trans..

[70] Hong D., Tsukakoshi Y., Kotani H., Ishizuka T., Kojima T. (2017). Visible-Light-Driven Photocatalytic CO_2_ Reduction by a Ni(II) Complex Bearing a Bioinspired Tetradentate Ligand for Selective CO Production. J. Am. Chem. Soc..

