# Supplementary material for: Sulphur vs NH Group: Effects on the CO2 Electroreduction Capability of Phenylenediamine-Cp Cobalt Complexes
Source: Molecules. 2023 Mar 4;28(5):2364. doi: 10.3390/molecules28052364 (PMC10005266; doi:10.3390/molecules28052364)
Supplement: Supplementary file 1 [file molecules-28-02364-s001.zip › molecules-2248707-supplementary.pdf]

SUPPORTING INFORMATION

Sulphur *vs* NH Group: Effects on the CO_2 Electroreduction Capability of Phenylenediamine-Cp Cobalt Complexes

Nicola Melis [1], Francesca Mocci [2], Annalisa Vacca [1] and Luca Pilia [1,*]

[1] Dipartimento di Ingegneria Meccanica, Chimica e dei Materiali (DIMCM), Università degli Studi di Cagliari, Via Marengo 2, 09123 Cagliari, Italy
[2] Dipartimento di Scienze Chimiche e Geologiche, Università di Cagliari, S.S. 554 km 4.500, 09042 Monserrato, Italy
* Correspondence: luca.pilia@unica.it

SUMMARY

Figure S1 – Plot of oxidation (red) and reduction (blue) **current densities** vs **(ν)$^{1/2}$** for complex **I**. . 3

Figure S2 – Graph **E_p-$E_{1/2}$** vs **ln(1/ν)** for derivative **II**. 4

Figure S3 – CV of $TBAPF_6$/DMSO 0.1 M under N_2 (solid blue line) and CO_2 (solid red line). CVs of complex **I** under the same conditions are reported for comparison (dotted lines)............................... 5

Figure S4 – CV of complex **I** at different scan rates. Current densities **J** are normalized by the square root of the scan rate. 6

Figure S5 – CVs of complex **II** at different scan rates.. 7

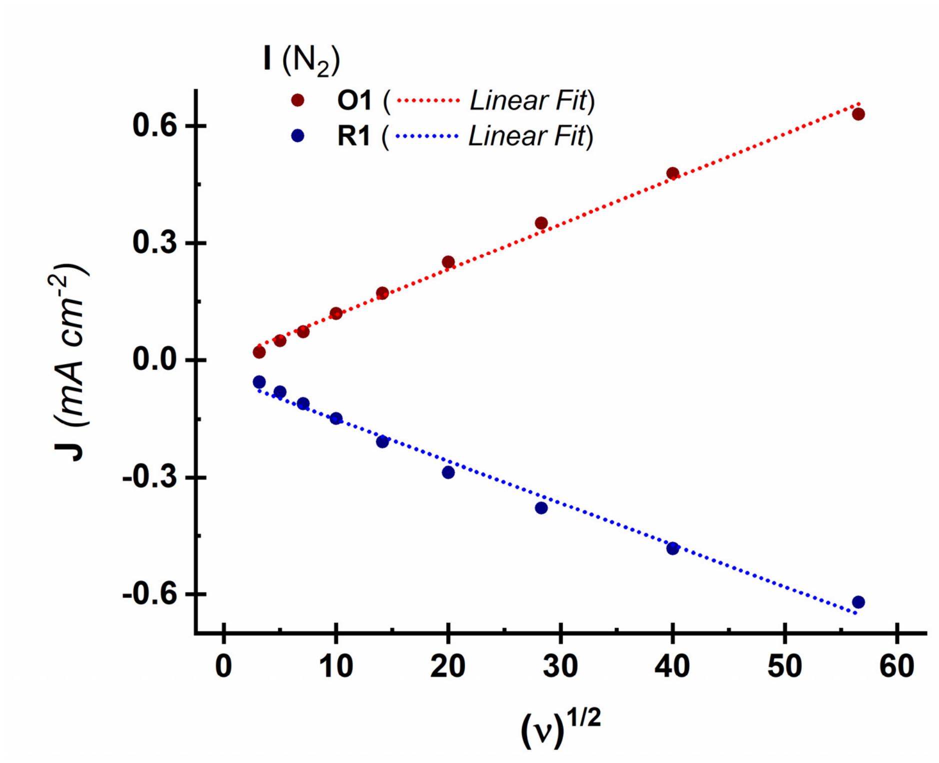

Figure S1 – Plot of oxidation *(red)* and reduction *(blue)* **current densities** *vs* **(ν)$^{1/2}$** for complex **I**.

KF CALCULATION

The rate constant $\mathbf{k_f}$ of the chemical step for compound **II** was determined using **eq S1**,[1] which correlates the potential shift occurring at different scan rates with the rate constant $\mathbf{k_f}$.

(Eq. S1)

$$\boldsymbol{E_p} = \boldsymbol{E_{\frac{1}{2}}} - 0{,}78\frac{RT}{nF} + \frac{RT}{2nF}\ln\left(\boldsymbol{k_f}\frac{RT}{nF}\right) + \frac{RT}{2nF}\ln\left(\frac{\mathbf{1}}{\boldsymbol{\nu}}\right)$$

Then, plotting the difference $\mathbf{E_p}$–$\mathbf{E_{1/2}}$ *vs* **ln(1/n)**, the value of the rate constant can be determined from the intercept of the linear fit. Data points for **ln(1/n) > 2** have

been excluded from the linear fit, as the reversibility is already reached. According to **eq. 1**, the plot in Figure S2 is expected to have a slope of 12.8 mV for single-electron processes. In the case of complex **II** , the observed slope is 13.4 ± 1.6 mV for scan rates ranged between 0.2 and 3.2 V s^{-1}; an intercept of 0.05883 led to a $\mathbf{k_f}$ of **1.90** $\mathbf{s^{-1}}$ and, consequently, a $\mathbf{t_{1/2}}$ equal to **364.8 s** for compound **II**.

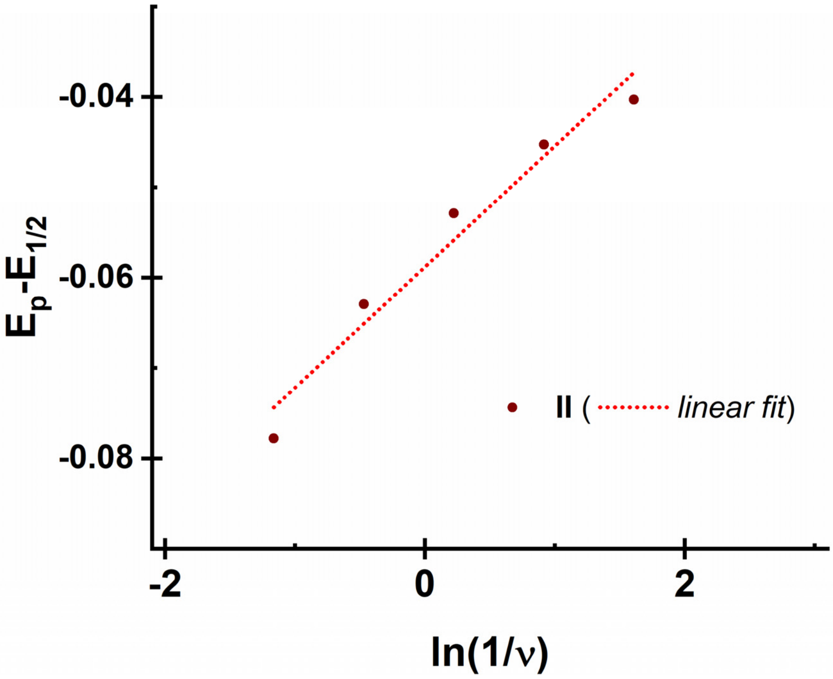

Figure S2 – Graph $\mathbf{E_p}$-$\mathbf{E_{1/2}}$ vs **ln(1/ν)** for derivative **II**.

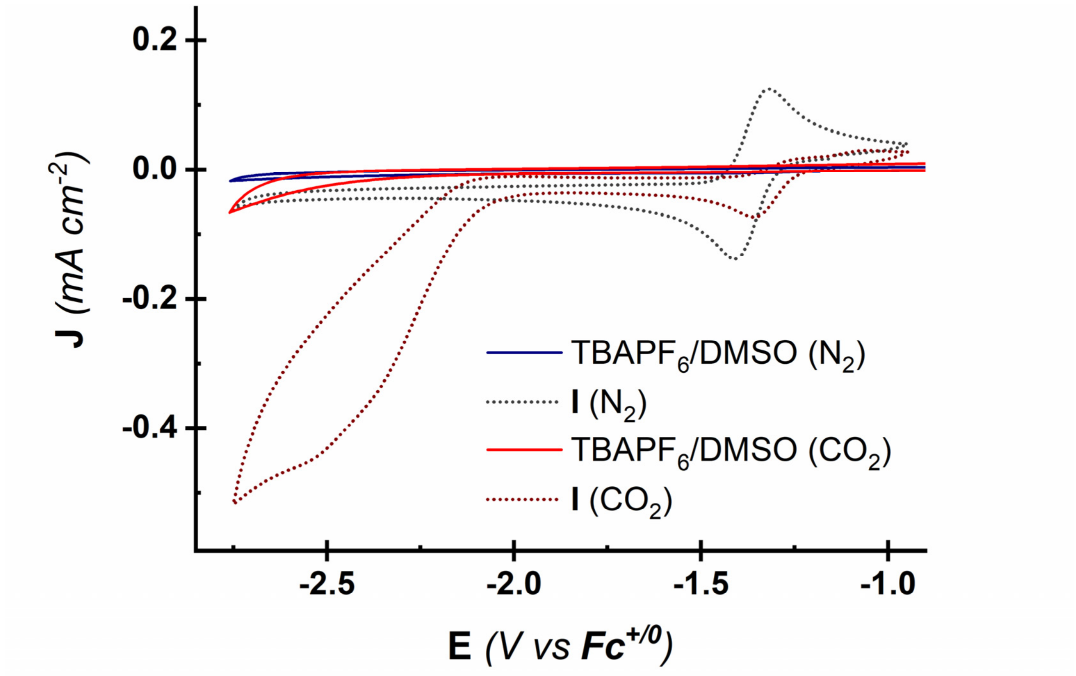

Figure S3 – CV of TBAPF6/DMSO 0.1 M under N_2 (*solid blue line*) and CO_2 (*solid red line*). CVs of complex **I** under the same conditions are reported for comparison (*dotted lines*).

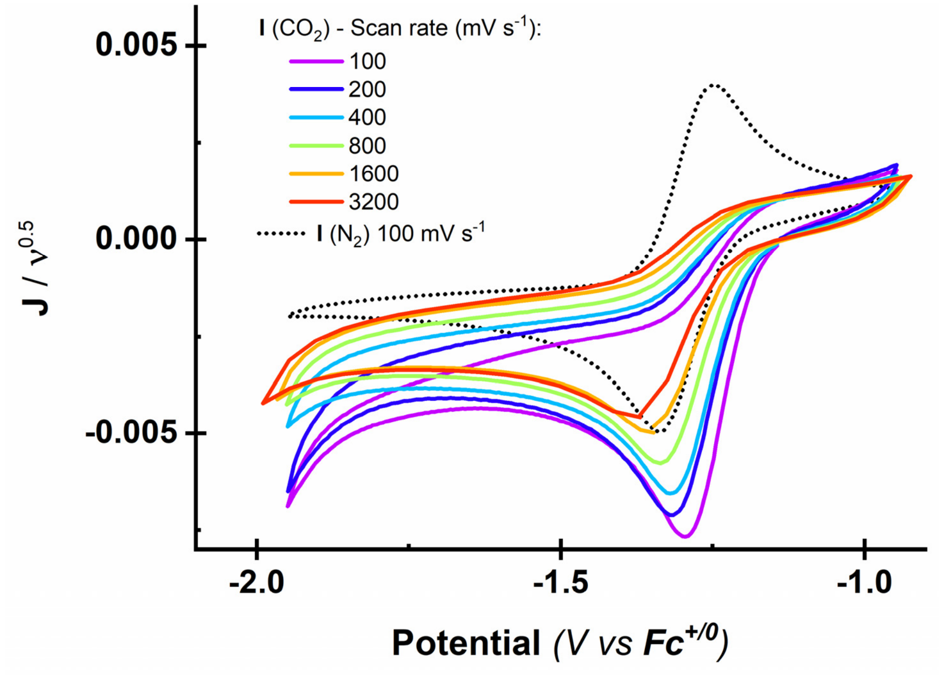

Figure S4 – CV of complex **I** at different scan rates. Current densities **J** are normalized by the square root of the scan rate.

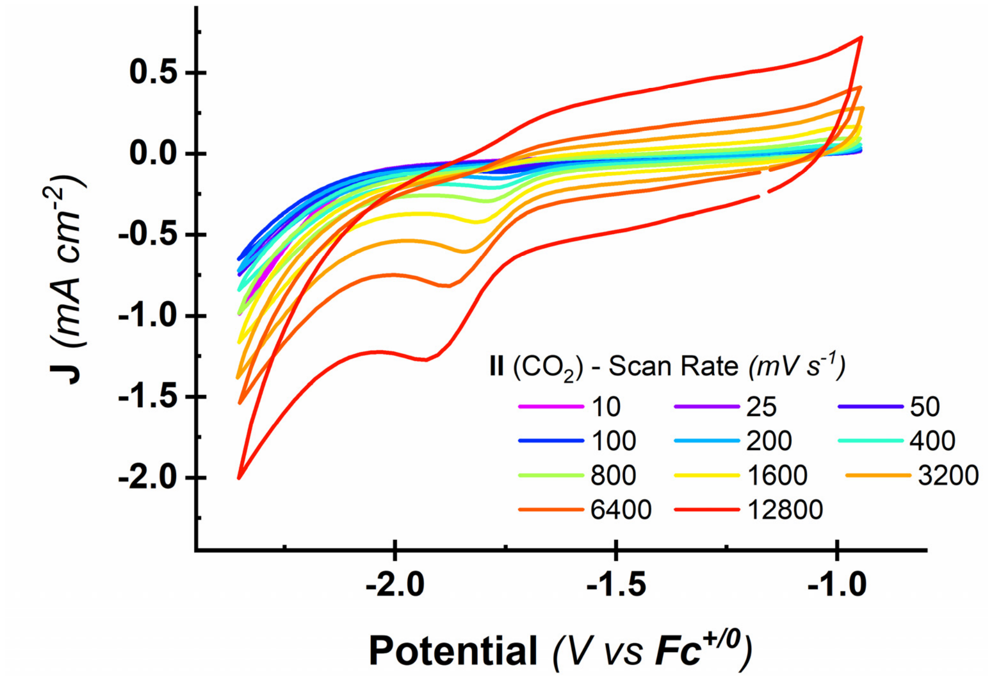

Figure S5 – CVs of complex **II** at different scan rates.

Reference

[1] R.S. Nicholson, I. Shain, Theory of Stationary Electrode Polarography, Anal. Chem. 36 (1964) 1212–1212. https://doi.org/10.1021/ac60213a053.
